# The underlying factors affecting the ethical performance of health service providers when faced with disasters: a qualitative study

**Published:** 2017-12-19

**Authors:** Mahmoud Abbasi, Mohsen Fadavi, Shabnam Bazmi

**Affiliations:** 1 *Associate Professor, Medical Ethics and Law Research Center, Shahid Beheshti University of Medical Sciences, Tehran, Iran.*; 2 *PhD Candidate in Medical Ethics, Department of Medical Ethics, School of Traditional Medicine, Shahid Beheshti University of Medical Sciences, Tehran, Iran.*; 3 *Associate Professor, Department of Medical Ethics, School of Traditional Medicine, Shahid Beheshti University of Medical Sciences, Tehran, Iran.*

**Keywords:** *Health service providers*, *Professional ethics*, *Disasters*, *Ethical performance*

## Abstract

Disasters are sudden catastrophic events leading to decisions in health service provision that are not in compliance with the principles and frameworks used in normal circumstances. It is essential to develop guidelines in order to ensure the ethical performance of health service providers and to prevent and manage the adverse consequences. As the first step in guideline development, the present study investigated the underlying factors affecting the ethical performance of health service providers in disasters.

This was a qualitative research based on grounded theory, and was conducted through unstructured in-depth interviews with various health service providers including paramedics, physicians and crisis zone managers who had some experiences in a number of domestic and foreign disasters. The collected data were analyzed using conventional content analysis.

The underlying factors extracted from the 24 interviews were divided into structural and mediatory factors. The structural factors covered the nature of the disaster, the type of social interactions, and lack of a unity management; the mediatory factors were connected to the emotional atmosphere governing the field, the behavior of the local people, the locals’ economic status, the locals’ trust in the authorities, and the safety of the crisis zone.

We can look into more effective, continuous and dynamic relationships between the components of the process of ethical performance. It is evident, however, that the underlying factors have more effective roles than the other components. According to our findings, the role of the underlying, structural and mediatory factors are more of a threat than an opportunity in disasters.

## Introduction

Disasters are sudden catastrophic events that affect not only the public health, but also the society’s economic and social development (1). 

The survey of the Epidemiological Crisis Research Center of Disaster showed that the death rate in countries with low incomes is three times more than high-income countries. The increase in people's deaths in association with decreasing in the frequency of disasters shows the vulnerability of communities to natural disasters. Due to the disproportionate burden of disasters in low-income countries, there is a need to improve and develop measures to reduce harms (2).

 Of the 41 types of natural disasters reported around the world, 31 have occurred in Iran, making this country one of the ten most disaster-prone countries in the world (3). War, terrorism, airplane collision and nuclear incidents are conceived as man-made, or a combination of man-made and natural, disasters. As a country of geopolitical and geostrategic importance in the Middle East and Asia, Iran has always been exposed to all of the above-mentioned events.

When disasters strike, the decisions related to the provision of health services often do not comply with the principles and frameworks used in normal circumstances. Most disaster-prone countries face various ethical issues concerning health service provision, such as respect for autonomy regulation, implementation of useful medical procedures, human dignity of those engaged (including the people in the region and health care workers, etc.), precluding hurt to those involved, allocation of scarce resources, triage of the injured and victims, and information provision. Rios et al. believe that the challenging ethical principles which should be hold during these circumstances include fairness and equality-generating processes (4) 

Health care workers often face three basic challenges in disaster situations: accountability, decision-making restrictions and allocation of inadequate resources (5). Throughout crisis, we learn that the guidelines used in normal situations are often unfeasible because of the necessity of immediate decision-making, the emotional ambiance of the field and the shortage of resources. In addition, ambiguities emerge in the responsibilities and the ethical performance of health service providers in disasters (6). 

Previous studies propose that health care workers have an unclear conception of "personal preparedness" in disaster situations. Thus, it is essential to determine the major factors empowering people in such circumstances (7). National disaster guidelines need to be developed with an ethical approach for stake holders so that they can supply widespread health services in the face of disaster (8).

Iran is a vast country that is exposed to a variety of natural and man-made disasters, but is unfortunately lacking disaster guidelines based on professional medical ethics. The development of such guidelines is contingent upon determination of the factors affecting ethical performance and explanation of the exact process. The present article is based on a study conducted to determine the underlying factors affecting the ethical performance of health service providers in disasters in Iran.

## Method

The present qualitative study was conducted using a method that emphasized the understanding of human experiences as they are and in the context in which they have been lived (9).

This study performed based on grounded theory. The study began in spring 2014 and was completed in winter 2016. The research and the interviews took place in different cities in Iran and mostly in Tehran.

The participants were selected through purposeful and theoretical sampling among individuals with two attributes: 1) a theoretical knowledge of disasters, and 2) having the experience of presence in disaster zones. They consisted of relief workers with different roles and responsibilities, including paramedics, physicians, disaster managers, and transportation and evacuation coordinators. 

During the study, a total of 24 interviews were conducted. The first participant was chosen after careful assessment, and the next participants were selected based on the findings obtained from the previous interviews with the aim of deepening and expanding the findings. All the interviews were conducted by the same person (i.e., the corresponding author of this article). Each interview began with the general open-ended question “In your experience, what are the underlying factors affecting the ethical performance of health service providers in disasters?". The interviews were recorded and transcribed and simultaneously analyzed. Every interview lasted half an hour to 45 minutes. Data were collected through unstructured, in-depth, individual interviews that helped increase the depth of the information gained and the flexibility of the study. After each interview, the meaning units were extracted and the step-one category was defined. Then, this category was corresponded with the findings of the previous interviews, and thus the main categories were determined. Categories were directly extracted from the data (9), and sampling stopped with data saturation. Continuous comparative analysis and conventional content analysis were used to analyze the data obtained from human experiences. The following methods were used to increase the validity and rigor of the data: 1) prolonged engagement, 2) time triangulation, 3) peer check, peer debriefing and external check, and 4) choosing the principal participants (8).

**Ethical Considerations**

The participants submitted informed consents and were ensured of their right to withdraw from the study at any stage and to stop the recording of the interviews at their own discretion. They were also ensured of the confidentiality of their data and their anonymity throughout the study. Intellectual property rights and the privacy of the subjects were fully observed in the publication of the results. There were no conflicts of interest between the researcher and any legal or natural beneficiaries. The literature listed in the references was reviewed and cited with honesty and integrity. 

## Results

The participants consisted of 24 disaster relief workers with different roles. Four participants were female and the rest were male. Their mean age was 51 and their mean duration of involvement in disaster relief was 21 years. Some of the participants were volunteers and others were serving their organizational duties. The underlying factors obtained were divided into the structural and mediatory categories, as shown in Table1.

**Table 1:** The categories of underlying factors

Underlying Factors	Structural	Exclusivity and uniqueness of disasters
		Lack of organized communication
		Selection of service providers not based on professional competence
		Unorthodox interference of influential people
		The deficient education system
		Lack of a unity management
		Impaired social interactions
	Mediatory (Facilitator/Inhibitor)	Environmental attributes
		Personal attributes
		Demographic attributes
		Managerial attributes
		Public conduct

As shown in the table above, these two categories are also divided into sub-categories.

***“Exclusivity and uniqueness of the disaster” ***was one of the extracted subcategories of structural factors. Most participants believed that although they thought they had learned great lessons from the Bam earthquake, they did not have a satisfactory ethical performance in later events such as Varzaghan. They thought that these earthquakes might have been similar, but each had its own peculiarities. One participant commented: *[We should realize that each disaster has its own features and occurs only once]* (Participant No. 6).

***“*** ***Lack of organized communication*** ***”*** was another subcategory of structural factors. The participants attributed this problem to all intra- and inter-organizational levels. One participant said: *[Communication is not merely to convey information on the phone or on the transceiver, you should also know if the information is actually practical. And the type, time and means of communication need to be planned ahead] *(Participant No. 2). The majority of the participants believed that the improper exchange of information disrupts needs assessment and obscures the process of decision-making. This defect facilitates performance outside the principles of professional ethics. 

***“*** ***Selection of service providers not based on professional competence*** ***”*** was extracted as another subcategory. The participants unanimously believed that workers are recruited according to general conditions and then take classes that mainly focus on their technical performance. Some believed that in disasters that require large numbers of relief workers, the majority are volunteers with perhaps no proper training, which means a much higher likelihood of unethical performance. 

***“*** ***Unorthodox interference of influential people*** ***” ***was another finding of this study that may be due to various motivations such as "the need to be seen". One participant said: *[Occasionally, a director or an authority attends the zone just to get people’s votes in future elections and pretends to serve the victims by interfering in rescue operations. The presence of these opportunists can even make the relief workers leave the zone and stop providing services]* (Participant No. 3).

Some participants believed that the presence of managers and authorities with no disaster management duties often leads to delays in rescue operations and brings the rescue workers' spirits down.

***“*** ***The deficient education system*** ***”*** was obtained as a subcategory of structural factors. Most participants believed that they would have had at least some knowledge of ethics if issues related to ethical performance or ethical decision-making in disasters had been included in their training syllabus. They argued that there were no topics on what is nowadays construed as “the science of professional ethics in disasters” in any of their school programs. 

***“*** ***Lack of a unity management*** ***” ***was among the categories extracted in the study. The majority of the participants believed that although the position and activities of every organization are clearly defined in the guidelines, a unity management is nonexistent in practice. Rescue workers are thus faced with confusion and ambiguity, and may inadvertently perform actions contrary to professional ethics. One participant said: *[*"*If the workers are not properly managed, they make decisions according to their own knowledge, and disagreement, annoyance, exhaustion and tension may arise, which will make ethical performance unfeasible] *(Participant No. 4).

***“*** ***Impaired social interactions*** ***”*** was obtained as the last subcategory of structural factors. Most participants believed that public capacity is not fully taken advantage of in disasters as a result of improper communication with people and the inadequacy of announcements. Some participants said that the public should receive training in advance so that they know what services they can provide to themselves and to others in the face of disaster. They admitted that not only had they performed poorly in this respect, but had interacted ineffectively with people in times of actual disaster. The circumstances would then lead to conflict, which greatly affected the ethical performance of service providers and wore them out.

The mediatory factors were categorized into smaller categories, as shown in Table 2.

**Table 2:** The subcategories of mediatory (facilitator and inhibitor) factors

Mediatory (Facilitator/Inhibitor)	Environmental attributes	Existence of endemic diseases Unfavorable biogeographical conditions Vulnerable infrastructures
	Personal attributes	Inability to control one’s emotions Low threshold of tolerance Desire to be seen Inadequate training and experience
	Demographic attributes	Multiplicity of the population affected by the disaster The large numbers of vulnerable groups such as children and older adults Public culture Public awareness Locals’ economic status and poverty
	Public conduct	Unorthodox emotional behaviors Inappropriate expectations Unclear role of people in the rescue process Locals’ lack of trust in the authorities and action forces
	Managerial attributes	Lack of appropriate control over conflicting interests Inadequate attention to teamwork Ineffectual utilization of resources Improper maintenance Inappropriate distribution of resources

The participants also believed that deficiencies in or absence of public safety, outbreaks of endemic diseases and vulnerable infrastructures have been and still are among ***"the environmental factors"*** conducive to a performance contrary to professional ethics in health service providers.

Some participants believed that in the absence of public safety, health service providers are constantly faced with fear, concern and anxiety. They worry that they might harm themselves or damage the equipment they have been given.

Most participants believed that the existence of an endemic disease in the region could lead to a fear of illness among the service providers. One participant said: *[I later realized why some colleagues skived off work and preferred to work in support or warehouse rather than in the field and with people. They were afraid of catching malaria or leishmaniasis] *(Participant No. 10).

Another finding in the category of environmental factors was the vulnerability of the infrastructures. Some participants argued that when the staff saw the poor construction or management of the water and electricity supply networks and the buildings and constructions in the disaster zone, they came to the conclusion that the local people and authorities had not been concerned about their own safety and did not bother to resolve the issues, so why should relief workers try so hard and endanger their lives? They concluded that no matter how hard they tried, the result would be no good.

***“*** ***Personal attributes of the health service providers*** ***” ***was another factor that was extracted in the study. Most participants believed that health service providers currently lack the acceptable level or type of skills for teamwork in disaster situations. One participant said: *[Sometimes we feel we’re competing against each other, which is why instead of helping one another out as members of a larger group, we cause problems and hinder each other's work] *(Participant No. 7).

The ability to control one's emotions was another personal attribute that was considered significant. One participant commented: *[Many people come to help, but leave the very first night without notice when they see the conditions in the zone. Sometimes they don’t leave, but they are unable to work the following day due to worry and anxiety. Obviously, these people can’t have a proper ethical performance either] *(Participant No. 4).

Another finding pertaining to personal attributes was the health service providers' motivations. The majority of the participants believed that most people who come to help, whether from NGOs or official organizations, consider philanthropy to be their motivation; however, some of them come to serve in the disaster zone looking for adventure, or to market themselves, or simply to be seen. The participants believed that this group often lack an ethical performance and seek only their personal goals.

***“*** ***Demographic characteristics of the area*** ***” ***comprised another finding of the study. According to the participants, the locals’ economic status, their social culture, the public level of education, the population density and the large number of vulnerable groups directly or indirectly affect the ethical performance of the health service providers. 

Some participants argued that when a large number of people, including children and older adults, need extensive services, the workload and the associated mental pressure substantially increase and make relief workers more prone to unethical performance. 

Most participants believed that in some areas, people have specific behaviors that largely depend on their culture. For instance, they may be overly emotional or have their own particular social customs. If service providers are not aware of such details, their intervention may turn into a big challenge at the sign of the slightest conflict, and the people's cooperation may diminish as a result. 

Sometimes locals may not trust the authorities for different reasons and will not trust relief workers either and resort to lying to receive further services, and this adversely affects ethical performance. 

Another group of the participants pointed out that in the disaster zone, most people who have lived all their life in poverty tend to attempt to make up for part of this poverty when they see the help being sent to their zone, and this tendency makes them behave aggressively and have the wrong expectations; in these circumstances, the health service providers may be forced to act unethically. For example, when they see people raiding the water distribution vehicle when there is plenty of drinking water, they start throwing the boxes of water to the people. 

One participant explained: *[I have seen the other staff having to speak harshly and be aggressive or lie about not having so and so, or even refrain from distributing certain items, because despite understanding the crisis situation, they didn’t consider the people's reaction right] *(Participant No. 18).

***“*** ***Management factors*** ***” ***comprised another finding of this study. Some participants argued that improper management in the distribution, maintenance and utilization of resources leads to increased environmental pressure on health service providers and affects their performance. Most participants believed that failure to resolve the challenge leads to mental imbalance in everyone and severely affects their performance. In such situations, it is the management that can restore peace to the group and resolve the issue.

## Discussion

The present study is a qualitative study that was conducted using content analysis to identify and explain the underlying factors affecting the ethical performance of health service providers in disasters. 

Many experts in qualitative research believe that a number of mediatory (including preventive or inhibitor, and facilitating) and structural factors form the underlying aspects of phenomena. As noted in the results, attempts were made to enhance the validity and rigor of the data and form more comprehensive categories through the careful selection of participants. 

*“Exclusivity and uniqueness of disasters*” comprised an important finding of the study with subcategories including a) the uniqueness of each disaster, and b) the unfamiliarity of the environment. Each type of disaster, including natural and man-made (or technological), have its own distinct characteristics. This attribute creates major challenges in the ethical decision-making and performance of health service providers despite their utilization of all the necessary measures, knowledge, and experience. Chaos, disorder, disharmony and the mismatch of supply and demand are part of the nature of disasters and unexpected events. The failure to properly identify and control these components may lead to a performance contrary to professional medical ethics.

This uniqueness means that in addition to the general principles of disaster, there is a special and unique situation in each case.

Communication disintegration was the main subcategory of *“lack of organized communication”*. This is a factor that paves the way for unethical performance through depletion of resources and exhaustion of rescue workers. A degree of communication failure occurs at all levels in disasters. Nevertheless, excessive disruption beyond the norm can affect the performance of any health service provider. Disaster zone management should base its plans and interventions on the information received from every point in the zone. Disruption in communication, both in terms of content and timing, makes planning incompatible with the needs of the target population, and may lead to unethical performance on the part of health service providers. In addition, the large groups of people looking into the disaster zone from outside also require correct and timely information for providing their support and empathy. This key role is highlighted in attracting people's support and the management of the post-disaster environment. 

It should be noted that in disasters, teamwork among people in a group or among groups is a moral obligation, and the existence of an organized link and the correct movement of information is an important part of this team work (10).

 It is imperative that information exchange be ongoing, accurate, documented, classified and timely. Obviously, in the absence of organized communications, we will witness parallel work and waste of resources, including financial and human resources.

The present findings regarding *“Selection of service providers not based on professional competence” *reveal the absence of a methodical system for assessing the professional competence of people as a key indicator of their performance in disasters. This reflects the defects in the present human resource recruitment system and the unorthodox focus of planners on having the maximum number of relief workers in disasters, which often leads to the attendance of poorly trained workers and obviously promotes unethical performance. 

Having the readiness, in addition to providing appropriate assistance to the injured, has other positive outcomes, including a greater willingness to engage in missions, and has a more favorable effect on recovery (11).

This care should prevail in both the initial recruitment of the forces and their training and cooperation.

*“Unorthodox interference of influential people” *was another finding of the present research that could be either a threat or an opportunity. Attendance of officials, directors and nationally or provincially influential people in the disaster zone when they have no predetermined or structural roles in the field can be very constructive if it accords with the disaster management goals and is free of personal, group, junta or faction interests. Their unprincipled conduct, however, often disheartens the health service providers and generates unethical performance, and may also lead to the misuse of the limited resources available, which is contrary to the ethical principle of equitable resource distribution. Influential people can engage in decisions within the scope of their responsibilities in executive centers outside the disaster zone, but they should realize that in the disaster zone, they have to follow the instructions and decisions of the zone manager.

*“The deficient education system” *was another finding of the study. The results indicated that medical ethics is not sufficiently covered in the curricula designed for disaster relief workers. Although the spirit of philanthropy governs the provision of health services in disasters, lack of ethical knowledge is conducive to a performance contrary to professional medical ethics. An article pointed out a public health paradox in disasters as doubts in decision-making arise from lack of knowledge (12). It is obvious that education is considered as a very effective factor in the empowerment and competence of health care providers that facilitates the achievement of predetermined goals at times of disaster (13).

In order to make the right decision, it is imperative for the health care workers to have the knowledge, training, information and experience sufficiently (14).

Health providers are in many cases the first to help victims of disasters, and therefore educating them is very important. The objective should be to train them to evaluate, stabilize and treat patients with minimum specialized equipment. In addition, they must try to maintain their own health under the circumstances. 

*“Lack of a unity management” *was another important finding of the study that can result from a combination of factors such as the multiplicity of organizations in charge, a claimant management or trusteeship, and performance confusion between the relief workers and the security forces. Despite the instructions and guidelines available on the responsibilities of each organization in the event of a disaster, in practice, no authorities or agents are responsible for certain matters, while for some others, multiple organizations and institutions may claim responsibility. This set of circumstances is created through disorder, distrust, parallel efforts and depletion of resources, and paves the way for the unethical performance of any of the forces or even the organized groups. If the supply and use of human resources and equipment are carried out autonomously, regardless of the needs of the region and without coordination with other service providers, the emergence of redevelopment and parallel organizations and the consequential lack of coordination between them will greatly affect the delivery of fair services and the effectiveness of relief and rescue will damage.

*“Impaired social interactions”* was another finding that revealed the complexity of interventions in the society at times of disaster. In the absence of proper social interactions, guiding public interventions becomes an inefficient affair; this in turn leads to loss of the high public capacity for helping to search for, retrieve and rescue the wounded and victims and loss of their cooperation through creating feelings of ineffectiveness. They may even feel as if they are merely users of services and not part of the disaster management team, which then exacerbates the emotional atmosphere of mistrust and encourages the unprofessional performance of the health service providers. 

In fact, people in or nearest to the community of the disaster can generally provide the most valuable contributions. Each society has certain positive traits that are related to its identity, which can be used to manage emergency situations. 

"*The mediatory factors”* can be considered as the environmental factors that could lead to the unethical performance of health service providers. The lack of public safety is especially alarming for health service providers in disasters. Harm to themselves and damage to their equipment are valid threats both from the local people (who, due to the emotional crisis, are highly likely to exhibit aggressive and unorthodox behaviors) and from greedy individuals or even criminals who have entered the disaster zone for looting or other evil purposes. This feeling of unsafety affects the emotional status of health service providers and diminishes their ability to focus on their specialty and increases the likelihood of errors.

Health care workers are aware that communicable and other types of diseases can spread in disaster situations, and it is therefore imperative to ensure their own health. When service providers learn about an endemic disease in the region, they begin to behave differently. For instance, they try to perform activities that put them in the least contact with the diseased or the locals, which will naturally distance them from the people and may even make them avoid providing services to the people in need for fear of getting sick themselves. Although they are well informed about their duties, their fear leads to unethical performance. 

The importance of maintaining safety and avoiding hurting themselves by disaster-based health service providers has been mentioned as a deterrent to prevent these people from acting on their moral obligations. Contagious infectious diseases and security threats in the disaster area are among these risk factors (15).

Regarding the impact of defective and vulnerable infrastructures on ethical performance, the participants believed that the poor infrastructure of an area is indicative of the unsatisfactory performance of the region’s officials and the locals’ lack of demand. By this reasoning, they came to the conclusion that the people in these areas should expect to pay for their own indifference and not demand the sacrifice of the health care workers under the circumstances. This view occasionally leads to unethical performance in health service providers, for instance they make mistakes, do not take responsibility and thus disrupt teamwork.

*“Personal attributes” *of health care workers comprised a key finding of this study. People react differently to the various things that happen in a disaster. The participants revealed that some of the workers ran away from the scene, while others remained astounded for a while and then somehow get adapted to the circumstances. 

It must be accepted that a high level of stress and mental pressure, which can affect the quality and quantity of the services they provided, thereby suggesting a pressing need for providing relief workers with specific aids and further attending to their needs. Some participants considered lack of practical training as a reason for the low threshold of tolerance and the personal vulnerability of the forces present in the scene. Training does not make everyone equally efficient, and this difference in efficiency stresses the need for empowerment and ongoing learner assessment, so that the training can promote ethical performance and decision-making in crises. Experience was another finding of the study that has a key role and can be acquired through frequent involvement in collective events or through learning from other people's experiences (whether domestic or foreign). Step-by-step development is required for getting experienced; otherwise, unethical performance becomes more likely both in individuals and the workforce under their command.

As for the desire to be seen, it can be argued that people attend the disaster zone with different motivations, including philanthropy. However, those who enter the zone influenced by communal excitement or out of the desire to be seen and for pretense or for marketing purposes quickly wear out and often perform irresponsibly and unethically due to their psychological discontent.

The people's behavior, which mainly arises from their culture, can be emotional, demanding or imbued with unorthodox expectations, which are categorized in this study as *“demographic attributes”*.

Such behaviors provoke a reaction in health care workers that may appear unethical at first; for instance, they may restrict the distribution of the demanded items or cause the unbecoming allocation of resources as if they were given out of charity, or lead to the workers’ lying or yelling in order to control the collective behavior. One reason for unethical performance may be the multiplicity of the population, which entails enormous demands for services. Another reason is the large number of vulnerable groups, which causes physical and mental exhaustion not only because of the aforementioned reason, but also because of these people’s need for special attention. The locals’ poverty can also affect their behavior and lead them to take more than they need from the large amounts of donations brought to the area after the disaster. The reason is obviously their desire to compensate for their long-term suppressions and current disaster-induced suffering. Such motivations can lead to unconventionally demanding behaviors that can mar their adaptive interaction with the health service providers and in a way that causes reciprocal and non-interactive reaction on the part of others.

Donner et al. believe that the multiplicity and density of the population and the locals’ poverty exacerbate vulnerability in disasters (1). Fothergill and Peek also believe that poverty is fundamental in the perception of risk and the response to it (16). The results obtained in several previous studies emphasize the importance of the society’s degree of understanding, the empowerment of people, and the development of social capital for creating a community-oriented approach in disasters and emergency plans (17). Preparation and attention to the dominant culture ensures people that, when a crisis occurs, cultural considerations are carefully taken into account in the efforts made in the face of predicament, so that the most effective response can form and lives can be saved and pains alleviated (18). The issues discussed above do not justify the unethical performance of health service providers, but are realities that should be taken into consideration. 

*“Public conduct” *comprises unorthodox emotional behaviors, inappropriate expectations, the unclear role of people in the rescue process and the locals’ lack of trust in the authorities and action forces. It must be admitted that in severe catastrophic events such as the Bam earthquake, people feel tired, and their response may be a desire to receive maximum assistance. The numerous unconventional expectations may force the health care workers to show certain reactions in order to control the situation, which may then lead to conflict between the two parties and create delicate and vulnerable relationships. 

People do not have access to the necessary information for statistical calculations of the probability of harm, and obviously follow rumors, stories of others, and emotional accounts. 

An atmosphere of distrust in the authorities caused by recent or previous experiences can lead to harsh and angry behaviors and unorthodox and greedy expectations in people and thus cause the tensions discussed earlier. 

The absence of plans for determining people’s position in rescue activities as a great local force with a capacity to direct and reform the actions means dismissing an exceptional opportunity for comprehensive disaster management. 

*“Managerial attributes” *can directly and indirectly lower the rescuers’ threshold of tolerance and make them indifferent through creating a sense of dissatisfaction with service provision, exhaustion, a feeling of discrimination and intentional mismanagement, and all of these adversely affect the ethical performance of health care workers.

## Conclusion

According to the results obtained, there are two major groups of factors that can encourage a performance either in line with or contrary to professional medical ethics among health service providers in disasters, especially at the onset of the event. The first group of factors is completely dependent on the nature of the disaster, and a performance contrary to professional medical ethics is still likely in spite of anticipating all the steps of relief work and meeting all the needs arising in the disaster; in the case of these factors, preventive measures can merely reduce the risk. The second group of factors, however, relies on the forces’ preparation and planning, and the main flaw in ethical performance pertains to this group. The present findings discuss the most sensitive and vulnerable points that need to be urgently addressed in order to develop relevant ethical performance guidelines and management plans.

At the moment, we face a shortage of ethical knowledge about health in disasters, lack of inclusive plans related to professional ethics, and an absence of all-encompassing health management in disasters. In addition to all these problems, and issues related to the selection of strategies and their consequences, we should accept the operation strategies of the health care workers in disasters and face the challenges in professional ethics. According to our findings, the role of the underlying structural and meditative factors is more of a threat than an opportunity under the circumstances. 

In the absence of a roadmap and the proper guidelines for ethical performance, the diversity and multiplicity of evaluative tools can lead to personal and group performances contrary to medical professional ethics. Until the necessary guidelines are developed, two strategies can be adopted to resolve this constraint: first, the proper and careful selection of people for attending disaster zones, and second, increasing the ethical knowledge of health service providers.

## References

[1] Donner W, Rodríguez H Disaster risk and vulnerability: the role and impact of population and society.

[2] Anonymous The Human cost of natural disasters 2015, a global perspective..

[3] Khankeh H, Mohammadi R, Ahmadi F (2007). Health care services at time of natural disasters: a qualitative study. Iranian Journal of Nursing.

[4] Rios CL, Redlener M, Cioe E (2015). Addressing the need, ethical decision making in disasters, who comes first. Journal of US-China Medical Science.

[5] Kiani M, Fadavi M, Khankeh H, Borhani F (2017). Personal factors affecting ethical performance in healthcare workers during disasters and mass casualty incidents inIran: a qualitative study. Med Health Care Philos.

[6] Grimaldi ME (2007). Ethical decisions in times of disaster: choices healthcare workers must make. J Trauma Nurs.

[7] Wynia MK (2006). Ethics and public health emergencies: rationing vaccines. Am J Bioeth.

[8] Holt RG (2008). Making difficult ethical decisions in patient care during natural disasters and other mass casualty events. Otolaryngol Head Neck Surg.

[9] Corbin J, Strauss A (2008). Basics of Qualitative Research.

[10] Larkin GL (2010). Unwitting partners in death, the ethics of teamwork in disaster management. Virtual Mentor.

[11] Thompson C (2006). Making ethical decisions during disasters. Nurs N Z.

[12] O’Laughlin DT, Hick J (2008). Ethical issues in resource triage. Respir Care.

[13] Montan KL, Örtenwall P, Lennquist S (2015). Assessment of the accuracy of the medical response to major incidents (MRMI) course for interactive training of the response to major incidents and disaster. Am J Disaster Med.

[14] Karadag CO, Hakan AK (2012). Ethical dilemmas in disaster medicine. IRCMJ.

[15] Chaffee MW (2006). Making the decision to report to work in a disaster: Nurses may have conflicting obligations. Am J Nurs.

[16] Fothergill A, Peek L (2004). Poverty and disasters in the United States: a review of recent sociological findings. Natural Hazards.

[17] Sobelson RK, Wigington CJ, Harp V, Bronson BB (2015). A whole community approach to emergency management: strategies and best practices of seven community programs. J Emerg Manag.

[18] Bergeron WP (2015). Considering cultural in evacuation planning and consequence. J Emerg Manag.

